# A Treatise on the Chronic Inflammation and Displacement of the Unimpregnted Uterus

**Published:** 1864

**Authors:** 


A Treatise on the Chronic Inflammation and Displacement of the Unimpregnated Uterus. By Wm. H. Byford, A.M., M.D., Prof. of Obstetrics, &c., Chicago Medical College, Medical Department Lind University. Philadelphia: Lindsay & Blakiston. 1864. pp. 215.

Lest anything we might write concerning the above work of our colleague should be attributed to partiality, we prefer to copy the following notice from the *Cincinnati Lancet and Observer* :—

"We have received this little volume, sometime anticipated, from our old classmate with a great deal of pleasure and satisfaction. Its title perhaps sufficiently indicates the general scope of the work, and at the onset we might simply expect to find a treatise on the topics suggested, modified by the peculiar views, theories, and personal experience of the author. In some good degree such is the character of Prof. Byford's work, but it is something more besides.

"There are two parties who hold somewhat opposite and extreme views in uterine pathology. One party holds that the uterus has very little sympathetic influence in the system; that the diseases of the uterus are quite as often dependent upon affections of the other organs as of independent origin. Of course this class of pathologists believe that these general symptoms are to be relieved without particular attention to the local condition or treatment of the uterus itself. This is one extreme.

"The other party holds 'that the sexual system of the female, in a state of disease, exercises a very morbid influence over nearly the whole organization. That this morbid influence is particularly exerted over the spinal and cerebral nervous systems; and that the only sure and permanent relief is found in the cure of the disordered condition of the uterus.'

"Then we find still further that there are a variety of subdivisions in these partizan groups; thus we have a class of uterine pathologists who believe that all these sympathetic disturbances grow out of various degrees of inflammation and ulceration; another class of equally respectable authorities hold that the cause of these manifestations will almost always be found in some form or degree of displacement, and these maintain that the inflammation and ulceration are of but slight importance.

"Dr. Byford is one of those who not only believe in the great sympathetic influence of the uterus, but he is amongst those who especially believe 'that inflammation and its accompanying effects' are the conditions upon which its sympathetic energies depend.

"These explanations prepare the reader to anticipate in this little volume a vigorous exponent of the practice of local treatment as the important consideration in the management of uterine affections. In his introductory and general observations our author pursues the argument to some length, but we presume our readers will scarcely care for their synopsis. Perhaps, however, the most interesting and forcible point made is the parallel which he draws between the symptoms usually attendant on uterine disease and spermatorrhora; he gives a parallel tabular statement, and the similarity is certainly remarkable, and as Dr. Byford remarks, 'affords an argument in favor of the efficacy of local causes in producing uterine inflammation, and of the powerful and general sympathetic influence of them when once originated.'

"We cannot attempt a general review of the contents of this book, we only aim to convey an idea of its scope and tendency. One of the special excellencies of the book is its individuality. It gives very fairly a systematic account of the nature, causes, and plans of treatment of the diseases embraced in his field of observation, but he does not merely give them as an editor, he does not re-vamp and re-hash the prominent authorities; indeed you are at once impressed with the idea that authorities are kept out of sight, and the personal experience of the author is for the most part presented to you. There is of course a freshness in this style of book-making that is always acceptable to the practitioner. There is nothing in medical literature so greedily sought after, and read with so much gratification as the personal experiences and observations of respectable teachers; hence the rapidity with which works of clinical medicine, obstetrics, and surgery find a sale.

"Dr. Byford's book closes with a few illustrative cases, showing the results of strictly local treatment in cases of aggravated disease; they are only valuable however in connection with the detailed views embraced in the body of the book."

For sale by W. B. KEEN & Co. Price $2.00.

